# Mediating roles of self-esteem and positive childhood experiences in the relationship between problematic social media use and loneliness

**DOI:** 10.1186/s41155-025-00364-z

**Published:** 2025-10-13

**Authors:** Fırat Ünsal, Zafer Korkmaz, İlhan Çiçek, Nouf Abdullah Alshehri, Abdulmohsen Mohammed Abdullah Alkhulayfi, Murat Yıldırım

**Affiliations:** 1 Department of Psychology, Faculty of Science and Letters, Bitlis Eren University, Bitlis, Türkiye; 2 Department of Educational Sciences, Faculty of Education, Van Yüzüncü Yıl University, Van, Türkiye; 3 Department of Child Development, Health College, Batman University, Batman, Türkiye; 4 Department of Education and Psychology, University of Hafr Al Batin, Hafr Al-Batin, Saudi Arabia; 5 Department of Business Administration, Faculty of Economics and Administration, King Abdulaziz University, Jeddah, Saudi Arabia; 6 Department of Psychology, Faculty of Science and Letters, Ağrı İbrahim Çeçen University, Fırat Mahallesi Yeni Üniversite Caddesi No: 2 AE/1, Ağrı, 04100 Türkiye; 7 Psychology Research Center, Khazar University, Baku, Azerbaijan

**Keywords:** Social media use, Loneliness, Self-esteem, Childhood experiences

## Abstract

**Background:**

Problematic social media use has been linked to increased loneliness among university students, yet the mechanisms underlying this relationship remain unclear.

**Objective:**

The study examines the mediating roles of self-esteem and positive childhood experiences in the relationship between problematic social media use and loneliness among university students.

**Methods:**

A total of 464 university students aged 18 to 28 years (M = 22.71, SD = 2.71; 58% women) participated in the study. Data were collected via an online survey using standardized measures of positive childhood experiences, social media addiction, self-esteem, and loneliness.

**Results:**

The findings revealed a significant positive relationship between problematic social media use and loneliness, as well as significant negative associations between problematic social media use and both self-esteem and positive childhood experiences. Mediation analyses indicated that problematic social media use significantly predicted loneliness, accounting for 6% of its variance, while the combined influence of problematic social media use, self-esteem, and positive childhood experiences explained 37% of the variance in loneliness. Notably, both self-esteem and positive childhood experiences partially mediated the relationship between problematic social media use and loneliness.

**Conclusion:**

This study provides important evidence for designing and implementing interventions that aim to enhance self-esteem and foster positive childhood experiences to mitigate the negative effects of problematic social media use on loneliness.

## Introduction

The advance and consequent adoption of digital technology has fostered “social media” into a tool which has benefited day-to-day life by promoting social relations and personal growth (Ruben et al., 2021). Despite these advantages, social media also has a detrimental psychological impact, contributing to various mental health challenges. Problematic social media use (PSMU) has been identified as a significant factor linked to psychological issues (Andreassen et al., 2017; Çiçek et al., 2021, 2024; Green et al., 2024; Korkmaz et al., 2024; Shannon et al., 2022; Smith & Anderson, 2018; Yam & İlhan, 2020; Yıldırım & Türk Kurtça 2024; Yıldırım & Çiçek, 2022). Such overuse has been linked to impaired time management, sleep disturbances (Korkmaz et al., 2023), reduced productivity (Hawi & Samaha, 2016), heightened social isolation (Johnson & Smith, 2021; Karakuş & Tarhan, 2023), and increased prevalence of anxiety and depression (Demirci, 2019; Xue et al., 2025). Individuals frequently experience a compulsive urge to engage excessively with social media, negating its benefits and exacerbating PSMU (Caplan, 2017; Echeburúa & De Corral, 2010; Kwon et al., 2013).

Although the DSM-5 does not formally recognize PSMU, research has identified focus-like behaviors associated with it (Griffiths, 2013; Küçükvardar & Tıngöy, 2018; Marino et al., 2021). Moreover, studies reveal that both its negative psychosocial effects and its reported frequency are on the rise (Shannon et al., 2022; Smith & Anderson, 2018), indicating that PSMU is shifting from a mere behavioral concern to a significant public health challenge (Smith & Anderson, 2018).

A rather profound psychosocial issue related to PSMU is isolation, which emerges in circumstances where social connections are needed but not adequately fulfilled due to the absence of relationships or having qualitatively poor ones (Hawkley & Cacioppo, 2010). Loneliness encompasses feelings of disconnection, isolation, or social exclusion (Hawkley & Cacioppo, 2010) and frequently arises from inadequate or unsatisfactory social ties (Demirci, 2019; Johnson & Smith, 2021). In the digital era, loneliness has become increasingly prevalent, particularly among adolescents, young adults, and older adults (Çiçek, 2021; Güler & Yıldırım, 2025; Lin & Chiao, 2024; Shannon et al., 2022; Yıldırım, 2021; Yıldırım & Türk Kurtça, 2024).

Understanding the relationship between PSMU and loneliness is crucial for assessing how social media usage impacts psychological well-being (Arrivillaga et al., 2022; Yıldırım et al., 2023). Research consistently demonstrates that PSMU exacerbates feelings of loneliness (Hawi & Samaha, 2016; Wu et al., 2024) and negatively affects individuals’ quality of life (Bilgin & Taş, 2018; Çiçek et al., 2024; Korkmaz et al., 2023). The burden of these effects tends to differ from one age group to another, showing the importance of limiting social media use. The objective of this research is to understand how PSMU contributes to loneliness and, consequently, how these detrimental effects can be curbed.

## The mediating role of positive childhood experiences and self-esteem

Positive childhood experiences (PCEs) are increasingly recognized as potential protective factors in addressing PSMU. Research highlights that PCEs strengthen emotional responses, enhance psychological resilience, and positively impact adult health (Bethell et al., 2014; Ceri & Cicek, 2021; Kocatürk & Çiçek, 2021; Novilla et al., 2022; Şanli et al., 2024; Çiçek et al., 2024). By fostering psychological robustness, PCEs are thought to mitigate the negative effects encountered on social media platforms and reduce the likelihood of PSMU (Öztekin, 2024; Xue et al., 2025). Furthermore, PCEs are associated with promoting secure attachment, which enhances individuals’ capacity to form and maintain social relationships, thereby offering a buffer against adverse outcomes such as PSMU (Lee & Williams, 2013; Zimmer-Gembeck et al., 2017). Additionally, PCEs contribute to overall well-being by enhancing happiness and reducing the negative effects linked to social media addiction (Demir, 2021; Öztekin, 2024). Understanding the relationship between PSMU and PCEs can provide valuable insights into how individuals can establish a healthier balance on digital platforms and maintain their psychological resilience.

Self-esteem is another key protective factor in managing PSMU and its associated challenges, such as loneliness. Evidence suggests that individuals with high self-esteem are more resilient to the adverse effects of PSMU (Fatima & Bhatt, 2024; Hasırcı et al., 2023). High self-esteem facilitates the development of healthy social relationships and plays a critical role in reducing feelings of loneliness (Balcı et al., 2020; Landa-Blanco et al., 2024; Pezzi et al., 2024) and increases subjective well-being (Yildirim et al., 2019). Consequently, self-esteem emerges as a crucial factor in helping individuals manage social media’s negative psychological and social consequences. Understanding and fostering these protective factors—PCEs and self-esteem—can guide strategies to mitigate PSMU and promote psychological well-being, offering a path toward healthier engagement with digital platforms.

## Current study

**Fig. 1:**
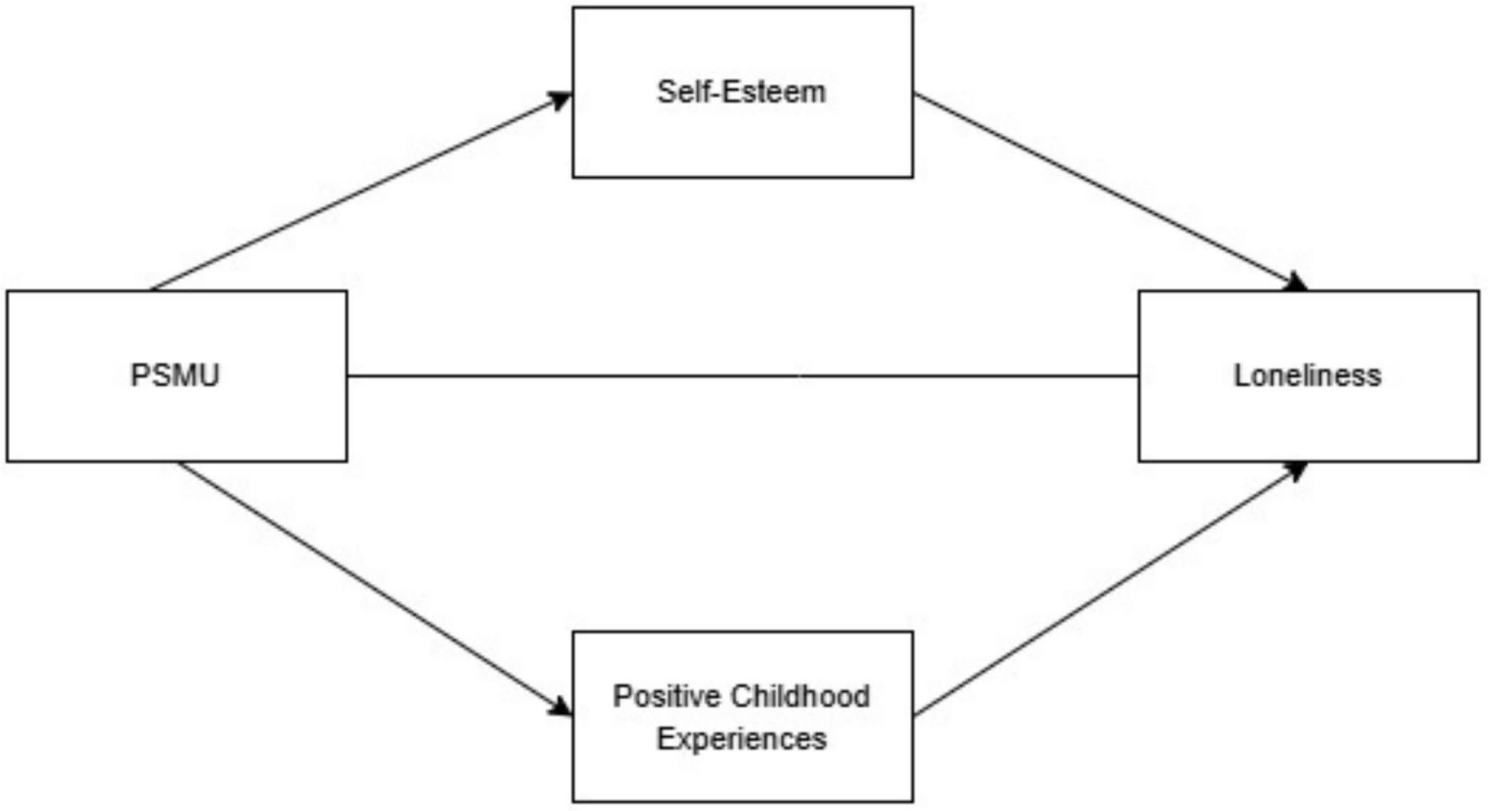
The proposed model of relationships between variables

This study aims to explore the mediating roles of self-esteem and PCEs in the relationship between PSMU and loneliness among university students. The intensification of social media use has shaped the way people interact, often fostering superficial connections while reducing the depth of meaningful relationships (Green et al., 2024). From a certain perspective, this creates a potential risk of increasing loneliness, particularly among users who exhibit problematic usage patterns. The study hypothesizes that PSMU increases loneliness levels, while self-esteem and PCEs act to buffer these negative effects. A systematic review of existing literature indicates that it is still very much the case: no one has investigated whether self-esteem and PCEs are themselves mediators of the relationship between PSMU and loneliness. This highlights the outlook of this research, which is relevant to developing new perspectives and encouraging research in this area. The focus of this study is guided by four main hypotheses that aim to clarify the intricate relationships of the construct of PSMU, loneliness, and its protective factors, such as PCEs and active self-esteem. (1) The first hypothesis states that there will be a significant and positive association between PSMU and loneliness, suggesting that excessive social media use may heighten feelings of isolation. (2) The second hypothesis predicts a negative relationship between PSMU and protective factors, indicating that higher levels of PSMU are associated with a decrease in PCEs, as well as self-esteem. (3) The third hypothesis explores the relationship between protective factors and loneliness, which suggests that PCEs and self-esteem are related to loneliness in a significantly negative manner. Finally, (4) the fourth hypothesis states that PCEs and self-esteem are the variables which PSMU affects indirectly. In this case, PSMU is expected to have a direct negative influence on the level of reported loneliness, but through the PCEs and self-esteem, these effects can be lessened. The proposed structural equation model for the formulation of the study (see Fig. 1) is designed to provide a more general explanation of the processes through which these protective factors can change the consequences of PSMU on loneliness. In these frameworks, one of the most important gaps in the literature will be filled, which is the lack of knowledge about factors that contribute to decreasing loneliness and increasing mental health among university students.

## Method

### Participants and procedures

The study group for this research comprised university students residing in Turkey, with a total sample of 464 participants (58% female, 42% male) aged between 18 and 28 years (Mage = 22.71, SD = 2.71). Participants were recruited using a convenience sampling method, especially targeting university students from various faculties and departments through social media networks and online students’ communities, and the sample size was determined using Monte Carlo Power Analysis, which indicated a minimum requirement of 310 participants for the mediation model (MacKinnon et al., 2004). The final sample size of 464 participants exceeded this threshold, ensuring sufficient statistical power.

The data was collected between September 10 and October 15, 2024, via social media platforms such as WhatsApp groups, X (formerly Twitter), and Instagram, using Google Forms. Participants were included if they were enrolled in a university in Türkiye, aged between 18 and 28 years, and provided informed consent, while those who failed to complete the survey, exhibited response bias, or did not consent were excluded. Ethical approval was obtained from the Batman University Ethics Committee (Ethics code: 2024/03-41). Participation was voluntary, with all participants providing informed consent for the use of their data for scientific purposes. To maintain confidentiality, responses were collected anonymously, and no identifying information was stored. Researchers followed all ethical guidelines for data protection, ensuring confidentiality and exclusive use for academic purposes. Inclusion criteria for the study: being a university student. Exclusion criteria: not being a university student.

### Measures

#### The Bergen Social Media Addiction Scale (BSMAS)

The scale is a 6-item instrument designed to measure problematic social media use (PSMU) based on six core addiction components: salience, mood modification, tolerance, withdrawal, conflict, and relapse (Andreassen et al., 2017). Each item is rated on a 5-point Likert scale ranging from 1 (very rarely) to 5 (very often), with total scores ranging from 6 to 30. Higher scores indicate more severe PSMU. A sample item is: “You think a lot about social media or plan how to use it.” The scale’s Turkish adaptation was conducted by Demirci (2019), and in the current study, Cronbach’s alpha reliability coefficient was found to be *α* = 0.80.

#### The UCLA Loneliness Scale (UCLA-8)

The scale is an 8-item short form of the original 20-item scale developed by Russell et al. (1978) to assess feelings of loneliness and inadequacy in social relationships. This single-dimension scale, widely used for measuring loneliness, was adapted into Turkish by Doğan (2011). Psychometric analyses confirmed its validity and reliability, with strong factor loadings and item-total score correlations. In this study, Cronbach’s alpha coefficient was calculated as *α* = 0.80, demonstrating its reliability.

#### The Self-Esteem Scale (SES)

The scale developed by Rosenberg (1965) and adapted into Turkish by Çuhadaroğlu (1986) assesses self-esteem levels through 10 items rated on a 4-point Likert scale (1 = strongly disagree to 4 = strongly agree). Higher scores signify higher self-esteem. In Çuhadaroğlu’s adaptation, Cronbach’s alpha was reported as *α* = 0.75, while in the current study, it was calculated as *α* = 0.84, confirming the scale’s reliability.

#### The Positive Childhood Experiences Scale (PCEs)

The scale, developed by Bethell et al. (2014), measures positive experiences individuals had before the age of 18. The scale consists of 7 items rated on a 5-point scale (1 = never, 5 = always), with total scores ranging from 7 to 35. Higher scores reflect more positive childhood experiences. Its Turkish adaptation and validation were conducted by Çiçek and Çeri (2021). In the current study, Cronbach’s alpha coefficient was found to be *α* = 0.79, indicating that the scale is a valid and reliable tool for the study population.

### Data analysis

This study explores the mediating roles of self-esteem and PCEs in the relationship between PSMU and loneliness among university students, employing structural equation modeling (SEM). SEM, a robust statistical method, facilitates the analysis of complex variable relationships while accounting for measurement errors (Kline, 2015). Before the main analyses, descriptive statistics, including means, standard deviations, skewness, kurtosis, and correlations were computed using SPSS (version 25). Pearson’s product-moment correlation analysis was conducted to examine the relationships among variables. Additionally, the PROCESS macro for SPSS (Model 4) was used to assess the mediating effects of self-esteem and PCEs on the relationship between PSMU and loneliness. Mediation effects were evaluated by estimating 95% confidence intervals (CI) based on 5000 bootstrap samples (Hayes, 2018). This comprehensive analytical approach provides critical insights into the factors influencing loneliness and PSMU within the study population.

## Results

Preliminary analyses confirmed that the study variables followed a normal distribution, with skewness values ranging from −0.42 to 0.64 and kurtosis values between −0.45 and 0.21, which fall within the acceptable range ( ≤|1|) for normality (Tabachnick & Fidell, 2013).

The correlation analysis revealed significant negative relationships between PSMU and both self-esteem (*r* = −0.21, *p* < 0.001) and PCEs (*r* = −0.20, *p* < 0.001). Additionally, PSMU was significantly and positively correlated with loneliness (*r* = 0.26, *p* < 0.001). Both self-esteem (*r* = −0.54, *p* < 0.001) and PCEs (*r* = −0.44, *p* < 0.001) were negatively and significantly associated with loneliness. The internal consistency reliability coefficients for the scales employed in the study were high, ranging from *α* = 0.79 to 0.84 (see Table 1). These results provide initial evidence for the reliability and validity of the measures used in this research.

**Table 1:** Descriptive statistics and correlation matrix

Variables	1	2	3	4
1. PSMU	1	0.26^**^	− 0.20^**^	− 0.21^**^
2. Loneliness		1	− 0.44^**^	− 0.54^**^
3. PCEs			1	0.40^**^
4. Self-esteem				1
Mean	16.07	14.68	24.85	30.16
SD	4.55	4.50	5.09	5.54
Skewness	− 0.424	0.649	− 0.392	− 0.355
Kurtosis	− 0.452	0.060	0.219	− 0.268
Internal reliability (*α*)	0.80	0.80	0.79	0.84

**p<0.001

### Mediation analysis

Following the preliminary analyses, we examined the mediating effects of self-esteem and PCEs on the relationship between PSMU and loneliness using the PROCESS macro (Model 4). The hypothesized model posits that PCEs and self-esteem mediate the influence of PSMU on loneliness.

**Fig. 2:**
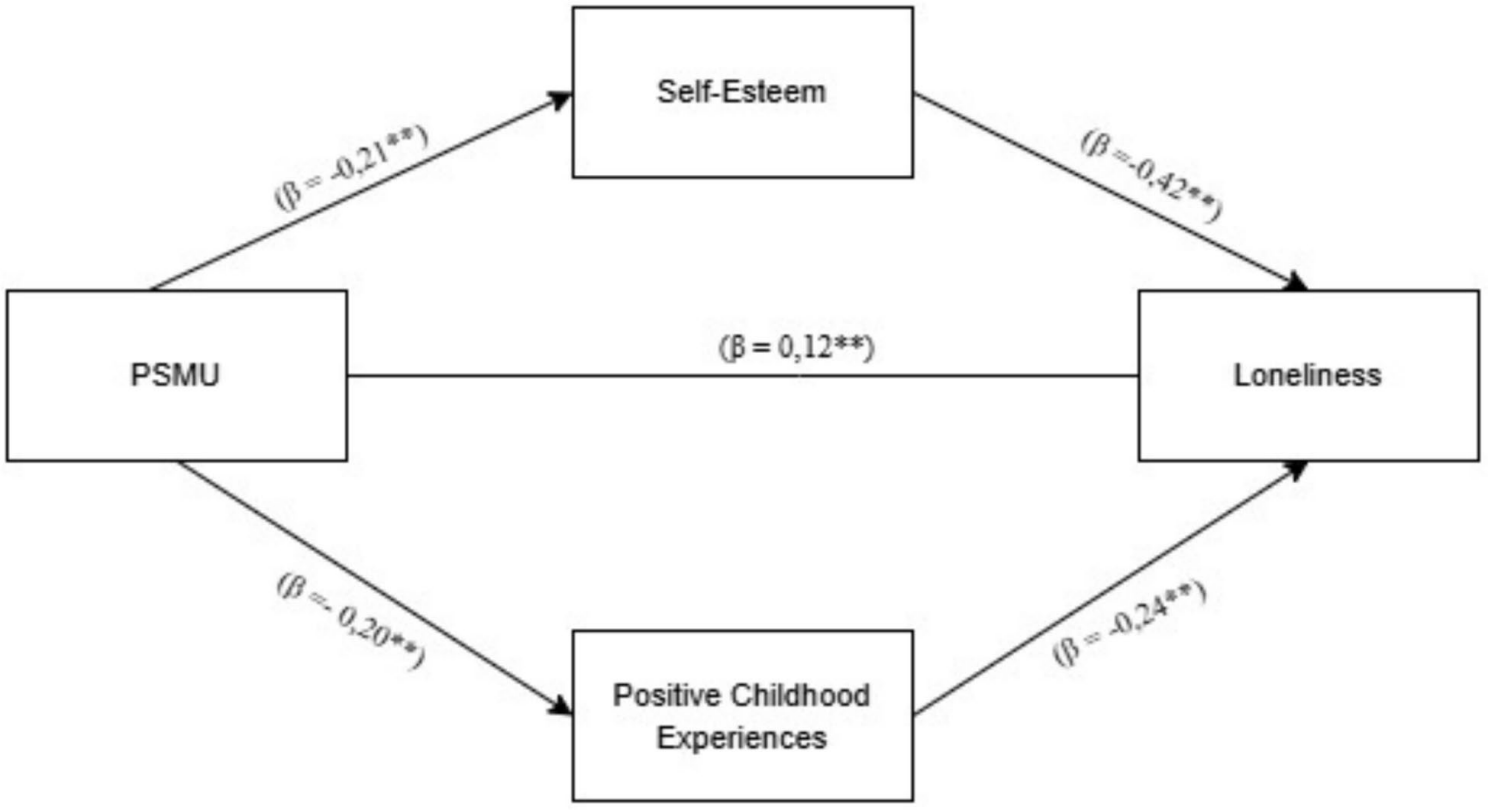
Mediation effect diagram of PCEs and self-esteem between PSMU and loneliness

As illustrated in Fig. 2, the findings revealed that PSMU significantly predicts loneliness in a positive direction (*β* = 0.12, *p* < 0.001). Conversely, both PCEs and self-esteem were found to significantly predict loneliness in a negative direction (*β* = − 0.20, *p* < 0.001; *β* = − 0.21, *p* < 0.001). Additionally, PCEs and self-esteem were negatively and significantly associated with loneliness (*β* = − 0.24, *p* < 0.001; *β* = − 0.42, *p* < 0.001).

The mediation analysis revealed that the effect of PSMU on loneliness was partially mediated by PCEs (effect = 0.05, 95% CI [0.02, 0.08]) and self-esteem (effect = 0.08, 95% CI [0.04, 0.13]) (see Table 2). These findings provide robust evidence for a significant parallel mediation effect, indicating that both PCEs and self-esteem partially mediate the relationship between PSMU and loneliness.

**Table 2:** PSMU, directly and indirectly, loneliness through PCEs and self-esteem

Path	Effect	Standard error	95% confidence interval	
			Lower limit	Upper limit
Direct effect				
PSMU → loneliness	0.11	0.377	0.452	0.193
Indirect effect				
PSMU → PCEs → loneliness	0.05	0.158	0.231	0.847
PSMU → self-esteem → loneliness	0.08	0.220	0.466	0.133
Total effect				
PSMU → loneliness	0.25	0.445	0.170	0.345

## Discussion

The findings of this study contribute to the growing body of literature on PSMU and its psychological implications, particularly in the context of loneliness. The primary aim of this study was to examine whether PCEs and self-esteem mediate the relationship between PSMU and loneliness. This aim was achieved, as the findings showed that the negative impact of PSMU on loneliness may be, at least in part, explained by diminished self-esteem and reduced exposure to positive childhood experiences. The emerging findings are discussed in accordance with the hypotheses of the study.

The first hypothesis proposes a positive association between PSMU and heightened loneliness. The findings confirm this relationship, aligning with existing literature. For instance, Marttila et al. (2021) conducted a longitudinal study examining the effects of PSMU on subjective well-being and the role of loneliness in this dynamic. Their findings confirmed that PSMU can elevate loneliness over time. Similarly, Karakuş and Tarhan (2023) demonstrated that excessive social media use exacerbates feelings of loneliness. Johnson and Smith (2021) observed that social media use amplifies social isolation and loneliness, particularly among young adults. Moreover, Erdemir and Ayas (2023) highlighted the association between PSMU, loneliness, and reduced life satisfaction. Kurt and Bayrakcı (2021) also reported that students with problematic social media habits experience higher levels of loneliness. Additionally, Yukay-Yüksel et al. (2020) concluded that social media use substitutes face-to-face interactions, thereby intensifying loneliness. These findings align with our research, reinforcing the negative impact of PSMU on loneliness.

The second hypothesis proposes that PSMU has a significant negative impact on self-esteem and PCEs. The findings support this assertion; however, existing literature on this relationship remains scarce, highlighting the need for further research. Andreassen et al. (2017) emphasized a significant relationship between social media addiction and low self-esteem. Similarly, Balcı et al. (2020) explored the bidirectional effects of social media addiction and self-esteem, revealing that problematic use diminishes self-esteem. Although studies on PCEs exist (Willis et al., 2024), no research directly examines the link between PSMU and PCEs. Most existing literature focuses on the adverse impact of negative childhood experiences on life outcomes (Bethell et al., 2014; Çiçek & Çeri, 2021; Lin et al., 2025).

The third hypothesis suggests a significant negative association between self-esteem, PCEs, and loneliness. Our findings indicate that individuals with higher self-esteem and more positive childhood experiences tend to experience lower levels of loneliness. In other words, as self-esteem strengthens and positive childhood experiences accumulate, feelings of loneliness diminish. This result is partially consistent with existing literature, reinforcing the importance of these psychological factors in mitigating loneliness. Many studies have documented a strong negative relationship between self-esteem and loneliness, showing that as self-esteem rises, loneliness diminishes (Sakız et al., 2021; Wrótniak, 2024). This relationship, particularly mediated by perceived social acceptance, is more evident in adolescents (Vanhalst et al., 2013). Individuals with high self-esteem are better equipped to manage loneliness through effective social communication; those with low self-esteem face an increased risk of loneliness, perpetuating a cycle of social isolation. Our research also underscores the effect of PCEs on loneliness. However, most studies in the literature focus on the detrimental effects of negative childhood experiences, with limited attention to the benefits of positive experiences (Lin et al., 2025; Lin & Chiao, 2024). Thus, interventions addressing both self-esteem and PCEs may be more effective in combating loneliness. Strategies that strengthen self-esteem and foster positive life experiences are essential in reducing loneliness.

The fourth hypothesis suggests that self-esteem and PCEs serve as mediators in the relationship between PSMU and loneliness, weakening this connection. The findings confirm this hypothesis, demonstrating that mitigating factors like self-esteem and positive childhood experiences can buffer against the detrimental effects of PSMU on loneliness. Furthermore, these results align with previous research, reinforcing the significance of these mediating mechanisms. For example, Andreassen et al. (2017) highlighted the role of self-esteem in social media addiction, noting that low self-esteem increases the likelihood of problematic use. Similarly, Hasırcı et al. (2023) found that self-esteem plays a critical role in the relationship between loneliness and PSMU. Our findings reveal that as self-esteem increases, the relationship between loneliness and PSMU diminishes. Furthermore, our results indicate that PCEs reduce the adverse effects of PSMU by alleviating loneliness. However, no studies in the current literature examine the mediating roles of PCEs and self-esteem in the relationship between PSMU and loneliness.

This study highlights the importance of protective factors in mediating the relationship between loneliness and PSMU. The consistency of our findings with existing literature enhances the reliability of the results and suggests the need for further research in different age groups and with larger sample sizes. Such studies could inform the development of intervention programs aimed at regulating social media use and reducing loneliness. Specifically, strategies focused on enhancing self-esteem and promoting positive childhood experiences may offer effective approaches to mitigating the impact of PSMU. This study aims to deepen understanding of this relationship and provide a foundation for future research and intervention initiatives.

### Limitations

This study has several limitations. First, the sample consists solely of university students; the findings are most relevant to this population and age group. The social media usage patterns and psychological characteristics of university students may differ from those of individuals in other age groups or socio-cultural contexts, suggesting that the results may apply only to this particular demographic. Additionally, the cross-sectional design of the study limits the ability to explore how the relationships between PSMU, loneliness, self-esteem, and positive childhood experiences evolve over time. Longitudinal studies are needed to assess the long-term effects of these variables. Furthermore, the measurement tools employed in this study may not fully capture cultural and contextual differences, highlighting the need for careful evaluation of the validity and reliability of the scales across diverse groups.

### Implications

The findings of this study present useful evidence about the impact of PSMU on loneliness and suggest strategies for mitigating these effects. The mediation of the relationship between PSMU and loneliness through self-esteem and positive childhood experiences may provide a framework for individuals to develop effective strategies for managing their social media use. Specifically, enhancing self-esteem and promoting positive childhood experiences could be key to alleviating the negative consequences of PSMU. Moreover, it is crucial to encourage individuals to reflect on their social media usage habits and consciously regulate the time spent on these platforms. Future research could expand our understanding of the effects of PSMU on psychological well-being by exploring these relationships across different age groups and cultural contexts. Such studies could lead to the development of more effective intervention strategies to manage PSMU and reduce loneliness.

## Conclusion

This study provides important evidence about the relationship between PSMU and loneliness, particularly in the context of self-esteem, positive childhood experiences, and their mediating roles among university students. The results indicate that PSMU contributes to loneliness, which, in turn, exacerbates psychological issues. Especially, PSMU fosters social relational disconnection, making it more difficult for individuals to engage socially, thereby intensifying feelings of loneliness. However, the study also identifies self-esteem and positive childhood experiences as mediators for this relationship. Inclusively, people who seem to be better off in interactions are also said to have greater self-efficacy, and positive childhood experiences appear to be less susceptible to PSMU’s negative impacts. This suggests that self-esteem and positive childhood experiences serve to strengthen an individual’s response to these sources of energy and tension, therefore reducing the effects of negative social media use. Ultimately, fostering self-esteem and promoting positive childhood experiences may help mitigate the psychological and social consequences of PSMU among university students, improving their overall well-being. Based on these findings, we recommend strategies to strengthen these protective factors in the student population.

## Data Availability

Data sets generated and analyzed in this study are available from the corresponding author upon reasonable request.

## Abbreviations

PSMU: Problematic social media use
DSM-5: Diagnostic and Statistical Manual of Mental Disorders, Fifth Edition
PCEs: Positive childhood experiences
BSMAS: Bergen Social Media Addiction Scale
UCLA-8: UCLA Loneliness Scale
SES: Self-Esteem Scale
SEM: Structural equation modeling
CI: Confidence intervals
M: Mean
SD: Standard deviation
